# Prevalence of Anemia and Its Associate Factors among Women of Reproductive Age in Lao PDR: Evidence from a Nationally Representative Survey

**DOI:** 10.1155/2021/8823030

**Published:** 2021-01-15

**Authors:** Sengtavanh Keokenchanh, Sengchanh Kounnavong, Akiko Tokinobu, Kaoru Midorikawa, Wakaha Ikeda, Akemi Morita, Takumi Kitajima, Shigeru Sokejima

**Affiliations:** ^1^Department of Public Health and Occupational Medicine, Mie University Graduate School of Medicine, 2-174 Edobashi, Tsu-Shi, Mie 514-8507, Japan; ^2^Foreign Relation Division, Cabinet of the Ministry of Health, Rue Simeuang, Ban Thatkhao, Sisattanack District, Vientiane Capital, Laos; ^3^Lao Tropical and Public Health Institute, Ministry of Health, Samsenthai Road, Ban Kaognot, Sisattanack District, Vientiane Capital, Laos; ^4^Department of Epidemiology and Preventive Medicine, Ehime University Graduate School of Medicine, 454 Shitsukawa, Toon-shi, Ehime 791-0295, Japan; ^5^Faculty of Child Education, Suzuka University, 663-222 Koriyama-Cho, Suzuka-Shi, Mie 510-0298, Japan; ^6^Epidemiology Centre for Disease Control and Prevention, Mie University Hospital, 2-174 Edobashi, Tsu-Shi, Mie 514-8507, Japan

## Abstract

**Introduction:**

Anemia continues to be a major public health problem significant among women of reproductive age (WRA) in developing countries, including Lao People's Democratic Republic (Lao PDR), where the prevalence of anemia among women remains high. This study aimed to assess the prevalence of anemia and its associated factors among WRA 15–49 years in Lao PDR.

**Methods:**

We conducted a cross-sectional study, using the Lao Social Indicator Survey II, 2017 dataset. A total of 12,519 WRA tested for anemia were included in this study, through multistage sampling approaches. Binary logistic regression was used to determine the associated factors of anemia.

**Results:**

Of 12,519 women, 4,907 (39.2%) were anemic. Multivariate logistic regression revealed that living in central provinces (aOR: 2.16, 95% CI: 1.96–2.38), rural area (aOR: 1.1, 95% CI: 1.00–1.20), large family size with more than 6 persons (aOR: 1.14, 95% CI: 1.01–1.29), pregnancy (aOR: 1.46, 95% CI: 1.22–1.74), having any adverse pregnancy outcomes (aOR: 1.14, 95% CI: 1.03–1.25), poor drinking water (aOR: 1.24, 95% CI: 1.10–1.39), and poor sanitation facility (aOR: 1.15, 95% CI: 1.03–1.28) were significantly associated with an increased risk of anemia. Conversely, four factors were associated with anemia preventively, including being aged 25–34 years (aOR: 0.81, 95% CI: 0.74–0.90), postsecondary education (aOR: 0.76, 95% CI: 0.60–0.97), Hmong-Mien ethnicity (aOR: 0.48, 95% CI: 0.39–0.59), and watching television almost daily (aOR: 0.84, 95% CI: 0.75–0.95).

**Conclusion:**

Anemia continues to be a major public health challenge in Lao PDR. Interventions should be considered on geographic variations, improving safe water and sanitation facility, promoting of iron supplements during pregnancy, and health education through mass media for women in rural areas.

## 1. Introduction

Anemia is a condition of red blood cells insufficient to meet the body's physiological demands. According to the World Health Organization (WHO), the threshold hemoglobin (Hb) level for anemia is less than 120 g/l for nonpregnant women and 110 g/l for pregnant women aged 15 years and above [1]. Anemia is a global public health problem, with major consequences for human health as well as adverse impacts on social and economic development [2]. The WHO estimates two billion anemic persons and anemia is responsible for one million deaths a year, and about three-quarters of cases occur in Africa and Southeast Asia [3]. The global prevalence of anemia in pregnant and nonpregnant women was 38% and 29%, respectively, in 2011, translated to about half a million anemic nonpregnant women and 32 million anemic pregnant women. Worldwide, 50% of anemia cases are caused by iron deficiency, and other important causes, including infections, nutritional deficiencies, and genetic conditions [4].

Several studies had found various factors associated to increase the risk of developing anemia in women of reproductive age (WRA) [5–7] such as consumption of wells water as the sources of drinking water, having permanent sterilization, living in small and medium-sized cities, aged between 30–39 years, no or occasional smoking, spring and winter seasons, ethnicity, and having low education level. Moreover, older age, limited knowledge of anemia, pregnancy during the second and third trimesters, multiparity, and experience of abortions were more likely reasons of anemia in pregnant women and lactating women [5, 8–10].

Anemia is a crucial public health problem in Lao People's Democratic Republic (Lao PDR). Although the prevalence of anemia among pregnant and nonpregnant women was reduced from 56.5% to 36.0% and from 46.1% to 31.0% in 2006 and 2011, respectively [2, 11], it remained higher than the global average (29%) for nonpregnant women [12]. The improvement of anemia in the country might have been attributed to the program for intermittent iron and folic acid supplementation that targeted women of childbearing age [13]. However, there are few studies on anemia that have been conducted in Lao PDR, mostly focusing on preschool children [14] and adolescent women [15], and all of them were carried out on a small scale and limited to a specific region of the country. Moreover, the associated factors for the prevalence of anemia in WRA have not been investigated. Therefore, this study aimed to assess the prevalence of anemia and to identify its associated risk factors among WRA 15–49 years in Lao PDR.

## 2. Materials and Methods

### 2.1. Study Design and Data Sources

We used the data from the Lao Social Indicator Survey (LSIS) II, 2017, a nationally representative cross-sectional survey conducted in July to November 2017 by the Lao Statistic Bureau in collaboration with the Ministry of Health and the Ministry of Education and Sport, as part of the Global Multiple Indicator Cluster Survey (MICS) Program developed by the United Nations Children's Fund (UNICEF). It combines the MICS and the Demographic and Health Survey (DSH).

### 2.2. Sampling and Study Population

LSIS II used the multistage, stratified cluster sampling approach for the selection of the survey sample. The sampling frame for this survey consisted of a list of all villages, considered as enumeration areas (EAs) in the country. The version used as the sampling frame was the village register of December 2015. They were stratified by the type of residence (urban, rural with the road, and rural without road areas) in every 18 provinces, defined as sampling strata. A two-stage sampling unit was used to ensure that estimates were representative at the national level. At the first stage, 1,170 EAs (373 from urban areas, 687 from rural with road areas, and 110 from rural without road areas) were selected from each sampling strata by using systematic probability proportional to size sampling procedures. At the second stage, 20 households were randomly selected from each EA, resulting in a total of 23,400 households; more details were described elsewhere [16]. Anemia was tested for in WRA 15–49 years in half of the selected households from the general survey. On-site hemoglobin analysis was performed by using a battery-operated portable HemoCue analyzer to estimate the Hb level in grams per deciliter blood. WRA 15–49 years who were tested for Hb and had the result of anemia testing were included in this study The LSIS II data were collected based on the MICS6 model questionnaire by qualified and trained interviewers. Details of the questionnaires can be found in the report of LSIS II, 2017 [16].

### 2.3. Study Variables

The outcome variable was the presence of anemia during the survey, which is defined as Hb level below 11.0 g/dl and 12.0 g/dl for pregnant and nonpregnant women, respectively, in accordance with WH [1]. The independent variables included in the analysis were identified based on previous literature, including individual characteristics such as age, level of education, ethnolinguistic group, religion, health insurance coverage, marital status, pregnancy status (pregnant versus nonpregnant), and experience of adverse pregnancy outcomes for the last pregnancy; environmental and health-related factors, including exposure to mass media: reading newspapers/magazine, listening to radio, and watching television (TV); main sources of drinking water, type of toilet facility, tobacco/cigarette smoking, and alcohol drinking; and household factors such as the area of residence (urban, rural with road, and rural without road), the region of residence (northern, central, and southern provinces), household wealth status, and size of the family.

### 2.4. Data Analyses

We summarized anemia status in accordance with each independent variable by conducting descriptive statistics analyses. Pearson's chi-squared test was used to assess the association between anemia status and independent variables. A variable found statistically significant in bivariate analyses with *p*-values <0.05 was further analyzed in multivariate analysis, after checking for multicollinearity by examining the variance inflation factors (VIF), and only variables with VIF less than 2 were included. Binary logistic regression was performed to determine the associated factors of anemia among WRA. The results of univariate and multivariate logistic regression analyses were reported in crude odds ratio (OR) and adjusted odds ratio (aOR) with 95% confidence intervals (CI), respectively. Women's sampling weights were available in the dataset and were included in all analyses for adjusting the effects of the stratified cluster sampling approach. All data were analyzed on SPSS, version 25 (IBM Corp., Armonk, NY).

### 2.5. Ethics Statement

The study protocol was reviewed and approved by the National Ethics Committee for Health Research, the Ministry of Health, Lao PDR (ID: 2020.33.NW). Verbal consent was obtained for each respondent participating prior to the data collection and each participant was informed of the voluntary nature of participation and the confidentiality and anonymity of information.

## 3. Results

### 3.1. Characteristics of Study Population and Prevalence of Anemia

A total of 12,519 WRA 15–49 years were included in this study. The mean age of the participant was 30.1 (±9.76) years. Around 35.1% of women had primary education and were living in the richest households (23.2%), with more than 6 people (24.5%). More than half of the women were Lao-Tai (65.1%), were married (73%), resided in rural with road area (57%), and had improved source of drinking water (85.9%) and improved sanitation facility (77.9%) (Table 1).

An overall prevalence of anemia among WRA15–49 years was 39.2% (95% CI, 38.3%–40%). The prevalence of anemia was particularly high among women who resided in the southern provinces (45.4%), were uneducated (42.3%), were married (43.6%), were pregnant (47.3%), experienced any adverse pregnancy outcomes (43%), ever smoked (42.1%), and had not watched TV at all (42%) (Table 1).

### 3.2. Associate Factors of Anemia

Multivariate analyses highlighted that women living in central provinces (aOR 2.16; 95% CI 1.96–2.38), southern provinces (aOR 2.05; 95% CI 1.82–2.31), and a rural area with the road (aOR 1.10; 95% CI 1.0–1.20) and with large family size with more than 6 people (aOR 1.14; 95% CI 1.01–1.29) were significantly associated with an increased risk of developing anemia as compared to those living in northern provinces and urban areas and with less than 4 household members, respectively. Similarly, pregnant women (aOR 1.46; 95% CI 1.22–1.74) and women who experienced any adverse pregnancy outcomes (aOR 1.14; 95% CI 1.03–1.25) had higher odds of developing anemia compared to their counterparts. In addition, increased odds of developing anemia were found among women who had an unimproved source of drinking water (aOR 1.24; 95% CI 1.10–1.39) and unimproved toilet facility (aOR 1.15; 95% CI 1.03–1.28). Conversely, compared to women aged 15–24 years, women aged 25–34 years (aOR 0.81; 95% CI 0.74–0.90) were negatively associated with developing anemia. Similarly, a low risk of anemia was found among women who had postsecondary education (aOR 0.76; 95% CI 0.60–0.97), belonged to Hmong-Mien ethnicity (aOR 0.48; 95% CI 0.39–0.59), and watched television almost daily (aOR 0.84; 95% CI 0.75–0.95) (Table 2).

## 4. Discussion

This study aims to assess the prevalence of anemia and to comprehend the influence of various factors, including individual characteristics and environmental and household factors on the prevalence of anemia among WRA 15–59 years in Lao PDR and to therefore identify its associated risk factors. This study revealed the prevalence of anemia among WRA remained high (39.2%). However, the prevalence of anemia among Lao women was lower than many other countries in the same region [17, 18], as well as developing countries on average [19]. However, it is still considered as a public health problem significant particularly among pregnant women as severe public health concerns, according to WHO classification [1]. Various associate factors for the prevalence of anemia were found after controlling for available independent variables.

At the individual level, we found that women who had any adverse pregnancy outcomes have higher odds of developing anemia. It is well documented that maternal anemia leads to poor neonatal outcomes such as low birth weight, stillbirth, preterm delivery, and early neonate death [20], women who had abortion reported a higher risk of developing anemia [8, 21]. Also, this might be due to iron deficiency [20, 22]. In Lao PDR, pregnant women usually receive iron supplementation during their antenatal care (ANC) visits. However, ANC visit rate in Lao PDR was increased from 35% to 54% in 2006 and 2011, respectively, but only half of those women took iron pills during their pregnancy [23]. Moreover, a high prevalence of insufficient iron intake (91%) was reported in a recent study [24]. In addition, current pregnancy shows an increased odd of anemia, this finding was found in other different settings [25–27]. Physiologically, the plasma volume increases progressively during pregnancy, causing a two-to three-fold and 10- to 20-fold increase in the requirement for iron and folate, respectively. For these reasons, the intake of iron and folic acid supplementation is necessary to maintain Hb at normal levels [28]. Therefore, interventions should highlight the promotion of iron and other micronutrient supplements during pregnancy.

On the other hand, our study found that women aged 25–34 had a lower risk of being anemic than those of younger ages. A similar finding was found in India [9]. As 60% of Lao people are younger than 25 years and early-age married, that resulted in early childbearing being very common among young women, with high birth rates at 15–19 years [29]. Women younger than 18 years had received less ANC visits [30]; consequently, this could be mean that these young women did not receive iron supplements during their pregnancy. Also, our study revealed that education was significantly associated with less risk of anemia among women who had postsecondary education compared to those who had no education. Several studies in different settings supported this finding [9, 31, 32].

Several environmental factors were found to be a positive risk of anemia. Unimproved sources of drinking water and unimproved toilet facility were about one time more likely to cause anemia as compared to the improved source of drinking water and sanitation facility. Naturally, arsenic enriched in groundwater had been found in tube well/hand pump drinking water supplies in Southeast Asia, including Lao PDR [33, 34], particularly in central and southern provinces of the country [35]. A previous study found an increased risk of anemia among women who had been exposed to arsenic from their drinking water [36, 37]. Although Lao PDR has made good progress in the provision of clean drinking water across the country, gaps still exist in rural villages [38], because 60% of the communities used dug wells as the main sources of water [39]. Moreover, Lao PDR had low rates of access to sanitation facilities in rural areas, where 73% of the population live [40]. Thus, we cannot neglect the probability of environmental contamination with parasites that causes anemia [41]. Therefore, interventions should strongly continue the provision of access to safe drinking water and improved sanitation facility in rural communities. Our findings were consistent with previous studies in other settings, including Myanmar [42], Nepal [6], Uganda [25], and Tanzania [26]. However, mass media exposure is a good source of receiving wider information, including health promotion programs, and increase chances of iron and folic acid supplements intake [43]. Our study showed that increasing the frequency of watching television is a significant protective factor against anemia in women; a similar finding was also found in India [44].

At the household level, geographic area of residence was found to be associated with anemia among women who lives in rural with road area and central and southern provinces who were one to two times more likely to be anemic, respectively. Similar associations between anemia and geographic location have been found in previous studies conducted in different settings of the same region [17, 27], and different region [6]. The southern provinces and some central provinces in Lao PDR are considered to be high malaria-endemic regions particularly in remote festered areas [45, 46]. The majority of malaria infections were asymptomatic and half of them were associated with anemia in women [47]. Nevertheless, the reduction of malaria confirmed cases more than 50% [48] and the high proportion of insecticide-treated bed nets coverage in endemic areas contributed to the reduction of malaria prevalence in Lao PDR [46, 49]; however, the effectiveness of treatment outcome for malaria by chloroquine, used as the first-line therapy for uncomplicated malaria and its resistance [50], and improper usage of insecticide-treated bed nets in rural areas during farming seasons [49] are a major challenge for malaria control in Lao PDR. Also, *Schistosoma mekongi* is an endemic parasite in a limited area of the southern province [51]. It is well documented that schistosomiasis infection is due to one of the parasites causing chronic blood loss, resulting in anemia [52, 53]. Even though schistosomiasis was selected for elimination by WHO, reservoir animals and insufficient safe sources of water in the endemic area make a control strategy challenging [51]. Thus, the interventions should be considering the malaria control strategies to ensure the availability and propriety of insecticide-treated mosquito bed nets, and deworming programs in affected geographic areas should be prioritized.

Also, our study found that a large family size with more than 6 people was significantly associated with an increase in the risk of anemia. A similar outcome was found in a previous study conducted in Ethiopia [54]. This might be due to food insecurity and distribution for large family size. In addition, access to foods is another issue, especially in a rural area, because less than 2% have permanent markets available in rural villages and poor quality road infrastructure, particularly during the rainy season which makes access to foods more challenging [38]. Moreover, a recent study in Lao PDR reported a high prevalence of insufficient diversity of food consumption (90.1%) and iron intake (61.8%) among WRA [24]. However, a low risk of developing anemia was found among women who belonged to the Hmong-Mien ethnolinguistic group. Geographically, this ethnolinguistic group mostly live in mountainous areas of the northern part of the country [38]. Physiological hemoglobin demands are greater among people living at high altitudes due to the low concentration of oxygen in the atmosphere [12]. Thus, the hemoglobin increases with an increase of altitude which is reported in a study conducted in Peru [55], particularly when the altitude is above 1,000 meters above sea level [56]. A study in Myanmar reported lower odds of anemia among women who lived in the hilly zone compared to those living in another zone of the country [27].

Our study has some limitations. Due to the cross-sectional nature of the survey, it is not possible to analyze the cause-effect relationship between anemia and the predictor variables. Also, this study was not able to assess other risk factors for anemia such as the family history of thalassemia, parasitic infections, contraceptive use, nutritional status, and micronutrient deficiencies such as folate, iron, and B-12, which might have potentially an important impact on the development of anemia.

## 5. Conclusions

The anemia continues to be a major public health challenge in Lao PDR. The prevalence of anemia among WRA was high (39.2%). Living in a rural area, in southern provinces, and with large family size, unimproved water and unimproved sanitation facility, being pregnant, and experiencing any adverse pregnancy outcomes were significantly associated to increase the risk of being anemia. Conversely, being aged 25–34 years and having postsecondary education and daily exposure to television were protective factors against anemia. Therefore, interventions should be considered on geographic variations, improving the provision of safe water and sanitation facilities, and promoting family planning and ANC by providing iron and acid folic supplements during pregnancy. Providing health education through mass media should be enhanced for women in rural areas.

## Figures and Tables

**Table 1:** Sociodemographic characteristics and prevalence of anemia among WRA in Lao PDR.

Characteristics	Total	Nonanemia	Anemia	*p*-value
	(weighted)	*n* (%)	*n* (%)	
**Age, years**	**12519**			**<0.001**
15–24	4285 (34.2)	2539 (59.3)	1746 (40.7)	
25–34	3911 (31.2)	2490 (63.7)	1421 (36.3)	
35–49	4323 (34.5)	2587 (59.8)	1736 (40.2)	

**Education**	**12519**			**0.02**
None	1996 (15.9)	1151 (57.7)	845 (42.3)	
Primary	4391 (35.1)	2696 (61.4)	1695 (38.6)	
Lower secondary	2726 (21.8)	1661 (60.9)	1065 (39.1)	
Upper secondary	1750 (14)	1067 (61)	683 (39)	
Postsecondary/nontertiary	418 (3.3)	272 (65.1)	146 (34.9)	
Tertiary	1238 (9.9)	770 (62.2)	468 (37.8)	

**Ethnolinguistic group**	**12518**			**<0.001**
Lao-Tai	8154 (65.1)	4843 (59.4)	3311 (40.6)	
Mon-Khmer	2878 (23)	1729 (60.1)	1149 (39.9)	
Hmong-Mien	1061 (8.5)	780 (73.5)	281 (26.5)	
Chinese-Tibetan	307 (2.5)	198 (64.5)	109 (35.5)	
Other	118 (0.9)	65 (55.1)	53 (44.9)	

**Religion**	**12518**			**<0.001**
Buddhist	8404 (67.1)	4993 (59.4)	3411 (40.6)	
Animist	3904 (31.2)	2501 (64.1)	1403 (35.9)	
Christian	186 (1.5)	108 (58.1)	78 (41.9)	
Other	24 (0.2)	13 (54.2)	11 (45.8)	

**Area of residence**	**12519**			**<0.001**
Urban	4202 (33.6)	2646 (63)	1556 (37)	
Rural with road	7140 (57)	4207 (58.9)	2933 (41.1)	
Rural without road	1177 (9.4)	763 (64.8)	414 (35.2)	

**Region of residence**	**12519**			**<0.001**
Northern provinces	3887 (31)	2842 (73.1)	1045 (26.9)	
Central provinces	6253 (49.9)	3474 (55.6)	2779 (44.4)	
Southern provinces	2379 (19)	1299 (54.6)	1080 (45.4)	

**Wealth quintiles**	**12519**			0.36
Poorest	2145 (17.1)	1268 (59.1)	877 (40.9)	
Poorer	2272 (18.1)	1381 (60.8)	891 (39.2)	
Middle	2414 (19.3)	1474 (61.1)	940 (38.9)	
Richer	2785 (22.2)	1727 (62)	1058 (38)	
Richest	2903 (23.2)	1766 (60.8)	1137 (39.2)	

**Size of family**	**12517**			**0.01**
≤3 persons	2040 (16.3)	1279 (62.7)	761 (37.3)	
4–6 persons	7409 (59.2)	4529 (61.1)	2880 (38.9)	
>6 persons	3068 (24.5)	1807 (58.9)	1261 (41.1)	

**Source of drinking water**	**12519**			**<0.001**
Improved	10760 (85.9)	6660 (61.9)	4100 (38.1)	
Unimproved	1759 (14.1)	956 (54.3)	803 (45.7)	

**Type of toilet facility**	**12518**			**<0.001**
Improved	9752 (77.9)	6094 (62.5)	3658 (37.5)	
Unimproved	2766 (22.1)	1521 (55)	1254 (45)	

**Health insurance status**	**12518**			0.06
Uninsured	10640 (85)	6436 (60.5)	4204 (39.5)	
Insured	1878 (15)	1179 (62.8)	699 (37.2)	

**Marital status**	**12519**			0.07
Never married	2826 (22.6)	1738 (61.5)	1088 (38.5)	
Currently married	9145 (73)	5569 (60.9)	3576 (39.1)	
Formerly married	548 (4.4)	309 (56.4)	239 (43.6)	

**Pregnancy status**	**12519**			**<0.001**
None	11961 (95.5)	7322 (61.2)	4639 (38.8)	
Currently pregnant	558 (4.5)	294 (52.7)	264 (47.3)	

**Adverse pregnancy outcomes**	**12519**			**<0.001**
No (live births)	10158 (81.1)	6261 (61.6)	3897 (38.4)	
Yes (stillbirths, miscarriage, abortion)	2361 (18.9)	1355 (57.4)	1006 (42.6)	

**Reading newspaper/magazine**	**12518**			0.13
None	10613 (84.8)	6420 (60.5)	4193 (39.5)	
Less than once a week	976 (7.8)	607 (62.2)	369 (37.8)	
At least once a week	718 (5.7)	463 (64.5)	255 (35.5)	
Almost daily	211 (1.7)	125 (59.2)	86 (40.8)	

**Listening to radio**	**12520**			0.27
None	9210 (73.6)	5571 (60.5)	3639 (39.5)	
Less than once a week	1118 (8.9)	710 (63.5)	408 (36.5)	
At least once a week	1141 (9.1)	692 (60.6)	449 (39.4)	
Almost daily	1051 (8.4)	643 (61.2)	408 (38.8)	

**Watching TV**	**12518**			**0.02**
None	2398 (19.2)	1395 (58.2)	1003 (41.8)	
Less than once a week	550 (4.4)	340 (61.8)	210 (38.2)	
At least once a week	1461 (11.7)	907 (62.1)	554 (37.9)	
Almost daily	8109 (64.8)	4973 (61.3)	3136 (38.7)	

**Ever smoked**	**12519**			**0.03**
None	11422 (91.2)	6981(61.1)	4441 (38.9)	
Yes	1097 (8.8)	635 (57.9)	462 (42.1)	

**Ever drank alcohol**	**12519**			**0.03**
None	2071 (16.5)	1217 (58.8)	854 (41.2)	
Yes	10448 (83.5)	6398 (61.2)	4050 (38.8)	

**Table 2:** Associate factors with anemia among WRA in Lao PDR.

Characteristics	Crude		Adjusted	
	OR (95% CI)	*p*-value	OR (95% CI)	*p*-value
Age, years				
15–24	Reference		Reference	
25–34	**0.83 (0.75–0.90)**	**<0.001**	**0.81 (0.74–0.90)**	**<0.001**
35–49	0.97 (0.89–1.06)	0.57	0.94 (0.85–1.04)	0.29

**Education**				
None	Reference		Reference	
Primary	**0.85 (0.76–0.95)**	**0.005**	0.89 (0.78–1.00)	0.06
Lower secondary	**0.87 (0.77–0.98)**	**0.02**	0.91 (0.79–1.05)	0.21
Upper secondary	**0.87 (0.76–0.99)**	**0.04**	0.91 (0.77–1.07)	0.26
Postsecondary/nontertiary	**0.73 (0.58–0.91)**	**0.006**	**0.76 (0.60–0.97)**	**0.03**
Higher	**0.82 (0.71–0.95)**	**0.001**	0.90 (0.75–1.08)	0.27

**Ethnolinguistic**				
Lao-Tai	Reference		Reference	
Mon-Khmer	0.97 (0.89–1.06)	0.51	0.94 (0.82–1.08)	0.45
Hmong-Mien	**0.52 (0.45–0.60)**	**<0.001**	**0.48 (0.39–0.59)**	**<0.001**
Chinese-Tibetan	0.80 (0.63–1.02)	0.07	1.23 (0.94–1.61)	0.11
Other	1.18 (0.82–1.71)	0.35	0.97 (0.66–1.41)	0.87

**Religion**				
Buddhist	Reference		Reference	
Animist	**0.82 (0.75–0.88)**	**<0.001**	1.04 (0.91–1.19)	0.49
Christian	1.05 (0.78–1.41)	0.72	1.14 (0.83–1.56)	0.41
Other	1.27 (0.57–2.82)	0.54	1.43 (0.63–3.25)	0.38

**Area of residence**				
Urban	Reference		Reference	
Rural with road	**1.18 (1.09–1.28)**	**<0.001**	**1.10 (1.00–1.20)**	**0.03**
Rural without road	0.92 (0.80–1.05)	0.24	0.90 (0.77–1.05)	0.21

**Region of residence**				
Northern provinces	Reference		Reference	
Central provinces	**2.17 (1.99–2.37)**	**<0.001**	**2.16 (1.96–2.38)**	**<0.001**
Southern provinces	**2.26 (2.03–2.51)**	**<0.001**	**2.05 (1.82–2.31)**	**<0.001**

**Size of family**				
≤3 persons	Reference		Reference	
4–6 persons	1.06 (0.96–1.18)	0.19	1.08 (0.97–1.20)	0.12
>6 persons	**1.19 (1.06–1.35)**	**0.004**	**1.14 (1.01–1.29)**	**0.03**

**Source of drinking water**				
Improved	Reference		Reference	
Unimproved	**1.36 (1.23–1.51)**	**<0.001**	**1.24 (1.10–1.39)**	**<0.001**

**Type of toilet facility**				
Improved	Reference		Reference	
Unimproved	**1.36 (1.25–1.48)**	**<0.001**	**1.15(1.03–1.28)**	**0.01**

**Pregnancy status**				
None	Reference		Reference	
Current pregnant	**1.41 (1.19–1.68)**	**<0.001**	**1.46 (1.22–1.74)**	**<0.001**

**Adverse pregnancy outcomes**				
No (live births)	Reference		Reference	
Yes (stillbirths, miscarriage, abortion)	**1.19 (1.08–1.30)**	**<0.001**	**1.14 (1.03–1.25)**	**0.008**

**Watching TV**				
None	Reference		Reference	
Less than once a week	0.86 (0.71–1.04)	0.12	0.84 (0.69–1.03)	0.10
At least once a week	**0.85 (0.74–0.97)**	**0.01**	**0.80 (0.69–0.92)**	**0.003**
Almost everyday	**0.87 (0.80–0.96)**	**0.006**	**0.84 (0.75–0.95)**	**0.005**

**Ever smoking**				
None	Reference		Reference	
Yes	**1.14 (1.00–1.29)**	**0.03**	0.99 (0.87–1.13)	0.94

**Ever drunk alcohol**				
None	Reference		Reference	
Yes	**0.90 (0.82–0.99)**	**0.03**	0.92 (0.83–1.03)	0.18

## Data Availability

The secondary data used in this analysis are available on reasonable request to the Lao Statistics Bureau, Ministry of Planning and Investment in Lao PDR. https://www.lsb.gov.la/en/home/
